# Serum alpha-fetoprotein and clinical outcomes in patients with advanced hepatocellular carcinoma treated with ramucirumab

**DOI:** 10.1038/s41416-021-01260-w

**Published:** 2021-02-03

**Authors:** Andrew X. Zhu, Richard S. Finn, Yoon-Koo Kang, Chia-Jui Yen, Peter R. Galle, Josep M. Llovet, Eric Assenat, Giovanni Brandi, Kenta Motomura, Izumi Ohno, Bruno Daniele, Arndt Vogel, Tatsuya Yamashita, Chih-Hung Hsu, Guido Gerken, John Bilbruck, Yanzhi Hsu, Kun Liang, Ryan C. Widau, Chunxiao Wang, Paolo Abada, Masatoshi Kudo

**Affiliations:** 1 Massachusetts General Hospital Cancer Center, Boston, MA USA; 2 Jiahui International Cancer Center, Jiahui Health, Shanghai, China; 3 Geffen School of Medicine, University of California, Los Angeles, Los Angeles, CA USA; 4 Asan Medical Center, University of Ulsan, Seoul, South Korea; 5 National Cheng Kung University Hospital, Tainan, Taiwan; 6 University Medical Center, Mainz, Germany; 7 Liver Cancer Program, Icahn School of Medicine at Mount Sinai, New York, NY USA; 8 Institut d’Investigations Biomèdiques August Pi i Sunyer, Hospital Clinic, University of Barcelona, Barcelona, Spain; 9 Department of Medical Oncology, CHU de Montpellier, Montpellier, France; 10 University Hospital S.Orsola-Malpighi, Bologna, Italy; 11 Aso Iizuka Hospital, Fukuoka, Japan; 12 National Cancer Center Hospital East-Hepatobiliary and Pancreatic Oncology, Kashiwa, Japan; 13 Azienda Ospedaliera Gaetano Rummo, Benevento, Italy; 14 Ospedale del Mare, Napoli, Italy; 15 Medizinische Hochschule Hannover, Hannover, Germany; 16 Graduate School of Medicine, Kanazawa University, Kanazawa, Japan; 17 National Taiwan University Hospital, Taipei, Taiwan, ROC; 18 Universtitätsklinikum Essen AöR, Essen, Germany; 19 Envision Pharma Group, Horsham, UK; 20 Eli Lilly and Company, New York, NY USA; 21 Eli Lilly and Company, Branchburg, NJ USA; 22 Eli Lilly and Company, Indianapolis, IN USA; 23 Kindai University, Osaka, Japan

**Keywords:** Oncology, Biomarkers

## Abstract

**Background:**

Post hoc analyses assessed the prognostic and predictive value of baseline alpha-fetoprotein (AFP), as well as clinical outcomes by AFP response or progression, during treatment in two placebo-controlled trials (REACH, REACH-2).

**Methods:**

Serum AFP was measured at baseline and every three cycles. The prognostic and predictive value of baseline AFP was assessed by Cox regression models and Subpopulation Treatment Effect Pattern Plot method. Associations between AFP (≥ 20% increase) and radiographic progression and efficacy were assessed.

**Results:**

Baseline AFP was confirmed as a continuous (REACH, REACH-2; *p* < 0.0001) and dichotomous (≥400 vs. <400 ng/ml; REACH, *p* < 0.01) prognostic factor, and was predictive for ramucirumab survival benefit in REACH (*p* = 0.0042 continuous; *p* < 0.0001 dichotomous). Time to AFP (hazard ratio [HR] 0.513; *p* < 0.0001) and radiographic (HR 0.549; *p* < 0.0001) progression favoured ramucirumab. Association between AFP and radiographic progression was shown for up to 6 (odds ratio [OR] 5.1; *p* < 0.0001) and 6–12 weeks (OR 1.8; *p* = 0.0065). AFP response was higher with ramucirumab vs. placebo (*p* < 0.0001). Survival was longer in patients with an AFP response than patients without (13.6 vs. 5.6 months, HR 0.451; 95% confidence interval, 0.354–0.574; *p* < 0.0001).

**Conclusions:**

AFP is an important prognostic factor and a predictive biomarker for ramucirumab survival benefit. AFP ≥ 400 ng/ml is an appropriate selection criterion for ramucirumab.

**Clinical Trial Registration:**

ClinicalTrials.gov, REACH (NCT01140347) and REACH-2 (NCT02435433).

## Background

Hepatocellular carcinoma (HCC) is the fourth leading cause of cancer-related death worldwide.^1^ Many clinical factors are important in the prognosis of patients with HCC, including disease stage, Eastern Cooperative Oncology Group performance status (ECOG PS), histopathology, liver function and serum alpha-fetoprotein (AFP) levels.^2–4^

Elevated AFP in patients with HCC is associated with worse prognosis compared with the general HCC population.^5–9^ It has been shown that incremental changes in AFP levels (10–<100, 100–<1000 and ≥1000) at the time of HCC diagnosis are significantly associated with increased mortality independent of several demographic factors, clinical factors or treatment.^6^ High serum AFP levels have been shown to predict the risk of tumour recurrence after hepatic resection and liver transplantation,^10–14^ and serum AFP concentrations ≥400 ng/ml have consistently been shown to indicate poorer prognosis in different clinical settings.^15,16^ Globally, patients with an AFP level >400 ng/ml comprise approximately half of HCC patients on systemic therapy.^5,17^

Vascular endothelial growth factor (VEGF) signalling through VEGF receptor-2 (VEGFR-2) plays a central role in angiogenesis, and blockade of VEGF/VEGFR-2 signalling is an important antiangiogenic strategy for cancer therapy. VEGFR is overexpressed in HCC and associated with poorer clinical outcomes,^18,19^ suggesting that VEGF-mediated signalling is important in HCC pathogenesis. Elevated serum AFP has been correlated with elevated VEGFR expression and increased angiogenesis.^20^ Evidence suggests that AFP expression may be associated with potentially more angiogenic tumours and could denote particular subclasses of HCC.^21^

Ramucirumab is a human IgG1 monoclonal antibody that specifically binds to the extracellular domain of VEGFR-2 with high affinity, preventing binding of the agonist ligands VEGF-A, VEGF-C and VEGF-D and, consequently, VEGFR-2 activation.^22^ Two placebo-controlled trials, REACH (NCT01140347) and REACH-2 (NCT02435433), have studied ramucirumab in patients with HCC after sorafenib, with REACH-2 enrolling only patients with baseline AFP ≥ 400 ng/ml.^17,23^ In REACH-2, ramucirumab improved overall survival (OS) and progression-free survival (PFS) compared with placebo in patients with HCC and elevated AFP without significant toxicity or compromise in patient-reported outcomes. This was consistent with REACH, in which a clinically meaningful improvement in OS was achieved in a prespecified subgroup of patients with baseline AFP levels ≥ 400 ng/ml treated with ramucirumab vs. placebo, even though significant improvement in OS with ramucirumab was not observed in the overall patient population.

In the current post hoc analyses, we assessed the prognostic and predictive value of baseline AFP, and we investigated the potential relationship between changes in AFP during treatment and outcomes of efficacy measures in the pooled population of patients with AFP ≥ 400 ng/ml, in both the REACH-2 and REACH studies.

## Methods

### Study design

Details of the REACH and REACH-2 studies have been described previously.^17,23^ Patients with advanced HCC, Barcelona Clinic Liver Cancer stage B or C disease that was refractory or not amenable to locoregional therapy, Child-Pugh A, ECOG PS 0–1 and with progression or intolerance to sorafenib were randomised in REACH (1:1) or REACH-2 (2:1) to receive ramucirumab 8 mg/kg or placebo every 2 weeks plus best supportive care until disease progression or unacceptable toxicity. In the REACH study, patients were enrolled irrespective of AFP level; in REACH-2, only patients with AFP ≥ 400 ng/ml were enrolled.

### AFP

Serum AFP levels were measured locally at baseline and every three cycles, that is, every 6 weeks until treatment discontinuation, and at short-term follow-up. AFP progression was defined as ≥20% increase from baseline and AFP response was defined as ≥20% decrease from baseline. Time to AFP progression was defined as the time from randomisation to AFP ≥ 20% increase from baseline. If no AFP progression or response was observed, the patient was censored at the last AFP assessment. Analyses of the per-patient AFP kinetics during treatment (from baseline to disease progression) were represented as the median percentage change in local AFP from baseline levels to AFP nadir (lowest AFP level up to the time of disease progression or end of study in the absence of disease progression) and from AFP nadir to disease progression for the pooled intent-to-treat (ITT) population of patients with AFP ≥ 400 ng/ml in the REACH-2 and REACH studies, and by the best overall radiographic response (complete response [CR]/partial response [PR], stable disease [SD] or progressive disease [PD]) by treatment arm.

### Statistical analyses

Associations between change from baseline AFP and efficacy endpoints, including radiographic progression, were analysed in the pooled population of patients with AFP ≥ 400 ng/ml in the REACH-2 and REACH studies. Time to progression (TTP) was defined as the time from randomisation to radiographic progression; radiographic response was assessed by protocol-defined criteria based on Response Evaluation Criteria in Solid Tumours version 1.1. If no radiographic progression was observed, the patient was censored at the last adequate tumour assessment. Overall response rate was defined as the proportion of patients who achieved CR or PR as their best overall response. OS was defined as the time from randomisation to death from any cause.

The prognostic value of baseline AFP was assessed by Cox regression models with either continuous or dichotomous (≥400 vs. <400 ng/ml) AFP (REACH, *N* = 565) and was validated using a Cox model with continuous AFP ≥ 400 ng/ml (REACH-2, *N* = 292). A Cox model of interaction between AFP and treatment arm assessed predictive value of baseline AFP in REACH.^17^

Subpopulation Treatment Effect Pattern Plot (STEPP) analyses were conducted in the ITT population of REACH and REACH-2: the subgroup of patients with AFP ≥ 400 ng/ml in REACH and a pooled population of patients with AFP ≥ 400 ng/ml in the REACH and REACH-2 studies. The purpose of the STEPP analyses was to determine whether the treatment effect changed for subpopulations with different baseline AFP values. STEPP analyses were conducted by dividing the overall patient population into overlapping subpopulations based on different thresholds of baseline AFP (known as a sliding window approach), estimating the treatment effect in each subpopulation, and plotting treatment effects against baseline AFP. Sliding windows were based on two cut-off values, AFP_min and AFP_max, which were chosen to have 100 patients in each window and 80 patients in common between two consecutive windows. Hazard ratios (HRs) were plotted against median AFP value in each window.

In addition, OS was evaluated by baseline AFP quartiles. Kaplan–Meier analyses of OS for baseline AFP quartiles in the pooled efficacy population in REACH-2 and REACH (AFP ≥ 400 ng/ml) were also conducted.

The baseline distribution of AFP level was plotted for comparison between arms for the pooled ITT population of patients with AFP ≥ 400 ng/ml in the REACH-2 and REACH studies. After taking log 10 of baseline AFP values, the frequency (patient count) was plotted for each arm.

Time to AFP progression and time to radiographic progression between treatment arms were evaluated by the Kaplan–Meier method. HR was generated using a stratified Cox proportional hazard model. AFP response rate is presented with 95% confidence interval (CI) and compared using the Cochran–Mantel–Haenszel test. AFP percentage changes observed in patients in the ramucirumab arm were compared with those in the placebo arm at cycles 3, 6, 9 and 12 by non-parametric Wilcoxon rank-sum tests. The association between the events of AFP progression and radiographic progression in each AFP measurement time interval was described by odds ratio (OR) and compared using Fisher’s exact test. All statistical analyses were done using SAS® Version 9.2 (SAS Institute, Cary, NC, USA).

## Results

### Predictive value of baseline AFP

Ramucirumab survival benefit for HCC patients with AFP ≥400 ng/ml was first observed in the prespecified subgroup of patients (AFP ≥400 ng/ml) in REACH, the first Phase 3 study of ramucirumab completed in patients with HCC. Post hoc analyses from REACH (Table 1 and Supplementary Fig. 1) showed a significant interaction between AFP and treatment effect, which suggested predictive value of AFP in OS benefit of ramucirumab treatment and a consistent OS benefit of ramucirumab treatment compared with placebo for patients with AFP ≥ 400 ng/ml.^17^

**Table 1:** Prognostic and predictive value of baseline AFP by Cox regression model.

Prognostic scenario	Hazard ratio (95% CI)^a^			
	REACH AFP dichotomous	REACH AFP continuous	REACH-2	Pooled
Adjusting for baseline AFP and treatment only^b^				
AFP (≥400 vs. <400 ng/ml)	1.93 (1.58–2.35), *p* < 0.0001	NA	NA	NA
AFP (ng/ml) log-transformed	NA	1.37 (1.27–1.47), *p* < 0.0001	1.59 (1.32–1.91), *p* < 0.0001	1.56 (1.37–1.76), *p* < 0.0001
Multivariate analysis^c^				
AFP (≥400 vs. <400 ng/ml)	1.82 (1.48–2.22), *p* < 0.0001	NA	NA	NA
AFP (ng/ml) log-transformed	NA	1.34 (1.25–1.44), *p* < 0.0001	1.58 (1.31–1.91), *p* < 0.0001	1.53 (1.35–1.74), *p* < 0.0001
ECOG PS (0 vs. 1)	0.77 (0.63–0.93), *p* = 0.0080	0.75 (0.62–0.91), *p* = 0.0040	0.68 (0.52–0.89), *p* = 0.0056	0.74 (0.61–0.90), *p* = 0.0020
Macrovascular invasion (yes vs. no)	1.49 (1.20–1.85), *p* = 0.0003	1.47 (1.18–1.82), *p* = 0.0005	1.44 (1.09–1.89), *p* = 0.0100	1.42 (1.17–1.73), *p* = 0.0004
Predictive scenario	Hazard ratio (95% CI)			
REACH AFP dichotomous^b^				
<400 ng/ml	1.06 (0.82–1.38), *p* = 0.664			
≥400 ng/ml	0.65 (0.50–0.85), *p* = 0.002			
AFP treatment interaction *p* value	0.008			
REACH AFP continuous^b^				
AFP treatment interaction (SE), *p* value	−0.195 (0.068), 0.0042			

*AFP* alpha-fetoprotein, *ECOG PS* Eastern Cooperative Oncology Group performance status, *NA* not available, *SE* standard error.

^a^All *p* values in prognostic scenario are Wald’s *p* values.

^b^Unstratified analyses.

^c^Adjusting for baseline AFP, treatment, macrovascular invasion and ECOG PS.

**Fig. 1:**
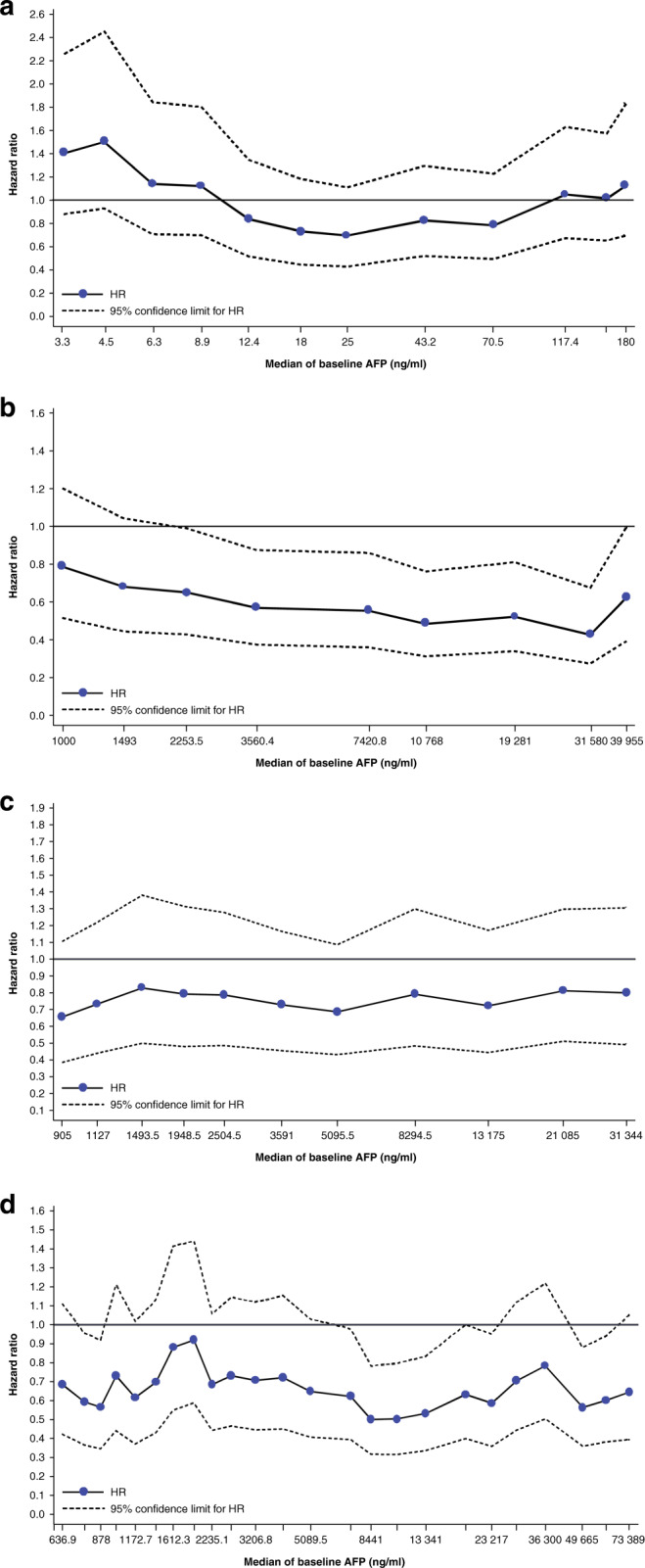
OS HR by baseline AFP. STEPP analysis showing OS HR by median baseline AFP in the **a** REACH study patients with AFP < 400 ng/ml (ITT population), **b** REACH study patients with AFP ≥ 400 ng/ml (ITT population), **c** REACH-2 study (ITT population) and **d** pooled population of patients with AFP ≥ 400 ng/ml in the REACH-2 and REACH studies. AFP alpha-fetoprotein, HR hazard ratio.

STEPP analysis was performed to show the median baseline AFP level against the OS HR for patients in subgroups defined by AFP levels in REACH. In the REACH study, the analysis observed fluctuations in the OS HR for subgroups with AFP < 400 ng/ml (Fig. 1a), but point estimates of the HR were consistently <1.0 for patients in subgroups with AFP ≥ 400 ng/ml (Fig. 1b), suggesting a consistent ramucirumab treatment benefit compared with placebo for patients with AFP ≥ 400 ng/ml. Together, these data informed the AFP selection criteria for the REACH-2 study, which enrolled only patients with AFP ≥ 400 ng/ml.

STEPP analysis in REACH-2 demonstrated a consistent OS benefit in all AFP subgroups, with point estimates for HRs consistently <1.0 (Fig. 1c). This result is further supported by analysis of the pooled efficacy population (patients in REACH-2 plus patients in REACH with baseline AFP ≥ 400 ng/ml), wherein the HRs were consistently <1.0 (Fig. 1d).

### Quartile analysis

Evaluation of OS by baseline AFP quartiles for patients in the pooled efficacy population supported the finding in the STEPP analysis, in showing that OS HR point estimates consistently favoured treatment with ramucirumab in all quartiles (Supplementary Table 1 and Supplementary Fig. 2).

### Prognostic value of baseline AFP

Consistent with historical data showing that an AFP level of ≥ 400 ng/ml defines a poorer prognostic group in several treatment settings, baseline AFP was confirmed as a significant continuous (REACH and REACH-2; *p* < 0.0001) and dichotomous (REACH; *p* < 0.0001) prognostic factor for OS (Table 1). AFP remained the predominant prognostic factor after adjusting for treatment and other significant prognostic factors, including macrovascular invasion, and ECOG PS.

### OS by AFP response

**Fig. 2:**
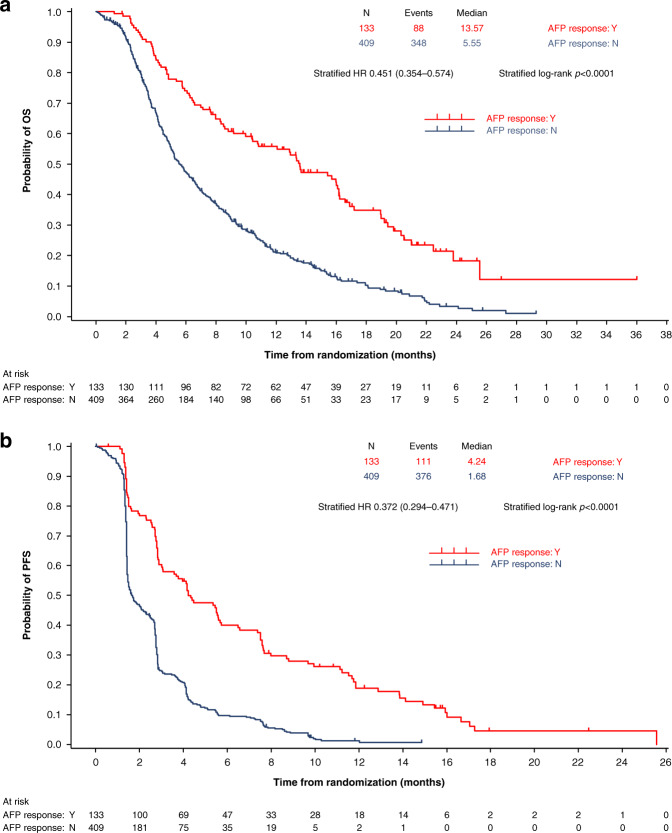
OS and PFS by AFP response. Kaplan–Meier graph of **a** OS and **b** PFS in patients with vs. without AFP response in the pooled population of patients with AFP ≥ 400 ng/ml in the REACH-2 and REACH studies. AFP alpha-fetoprotein, HR hazard ratio, N no, OS overall survival, PFS progression-free survival, Y yes.

Additional analyses on the relationship between AFP response and OS were performed. A Kaplan–Meier plot of OS for patients with either an AFP response (*n* = 133) or no AFP response (*n* = 409), irrespective of treatment arm, is shown in Fig. 2. The median OS for patients with an AFP response was significantly longer than that for patients without an AFP response (13.6 vs. 5.6 months; HR 0.451; 95% CI, 0.354–0.574; *p* < 0.0001). OS was significantly longer with ramucirumab compared with placebo among patients with no AFP response (6.7 vs. 4.8 months; HR 0.804; 95% CI, 0.650–0.994; *p* = 0.0436), but there was no significant difference in OS between treatments for those patients with an AFP response (13.6 vs. 12.1 months; HR 0.963; 95% CI, 0.523–1.772; *p* = 0.905).

As an AFP level >1000 ng/ml is commonly used to assess eligibility for liver transplantation in earlier settings,^24^ OS was assessed in patients with AFP 400–1000 and >1000 ng/ml. Median OS was longer with ramucirumab compared with placebo in patients with AFP 400–1000 ng/ml (11.1 vs. 7.6 months; HR 0.690; 95% CI, 0.443–1.077; *p* = 0.1003) and significantly longer in patients with AFP > 1000 ng/ml (7.7 vs. 4.6 months; HR 0.672; 95% CI, 0.540–0.835; *p* = 0.0003).

The median PFS among AFP responders was also significantly longer than that for AFP non-responders (4.2 vs. 1.7 months; HR 0.372; 95% CI, 0.294–0.471; *p* < 0.0001). In AFP non-responders, PFS was significantly longer with ramucirumab than placebo (2.3 vs. 1.5 months; HR 0.661; 95% CI, 0.535–0.818; *p* = 0.0001), but among AFP responders there was no significant difference in PFS between treatments (4.2 vs. 5.5 months; HR 0.768; 95% CI, 0.440–1.342; *p* = 0.3460).

### AFP response or progression

Changes in AFP during study treatment relative to baseline were assessed in the pooled population of REACH-2 and REACH (AFP ≥ 400 ng/ml). Patients were grouped and analysed by those with or without an AFP response during study treatment. Baseline and disease characteristics in the pooled population were generally similar between treatment groups and by AFP response (Supplementary Table 2). Median baseline AFP level for patients treated with ramucirumab was 3591 ng/ml for those with an AFP response, and 4473 ng/ml in those without an AFP response. The median baseline AFP level for placebo was 1853 and 4430 ng/ml for patients with and without an AFP response, respectively. A higher proportion of patients with hepatitis B virus at baseline was observed among AFP non-responders compared with AFP responders (46.0 vs. 28.6%), whereas the incidence of hepatitis C virus at baseline was higher among responders than non-responders (32.3 vs. 23.5%). In patients receiving ramucirumab, the median time on treatment was 19.4 weeks for patients with an AFP response (median nine cycles of treatment) and 8.0 weeks for those without an AFP response (median four cycles of treatment) (Supplementary Table 3). The median relative dose intensity for ramucirumab was 97.4% and 98.7% for patients with and without an AFP response, respectively.

**Fig. 3:**
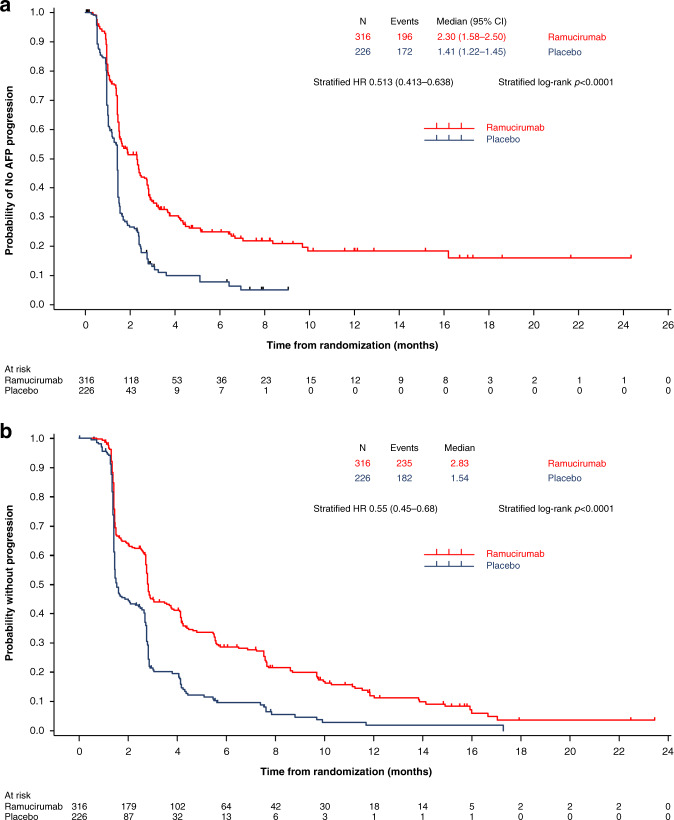
Time to AFP and radiographic progression. Kaplan–Meier plots of **a** time to AFP progression and **b** time to radiographic progression in the pooled population of patients with AFP ≥ 400 ng/ml in the REACH-2 and REACH studies. AFP alpha-fetoprotein, HR hazard ratio.

AFP response results for REACH have been reported previously.^25^ After log transformation of baseline AFP, the distribution of patients by log AFP for each treatment arm in the pooled population of patients with AFP ≥ 400 ng/ml in the REACH-2 and REACH studies appeared similar (Supplementary Fig. 3). Changes in AFP relative to baseline were analysed and defined as either AFP progression or response, or neither. A Kaplan–Meier plot of time to AFP progression for patients with AFP ≥ 400 ng/ml in the REACH-2 and REACH studies treated with ramucirumab vs. placebo is shown in Fig. 3a. The median time to AFP progression was 2.3 months in the ramucirumab arm and 1.4 months in the placebo arm, with an HR of 0.513 (*p* < 0.0001).

Consistent with the results on time to AFP progression, patients treated with ramucirumab were more likely to experience an AFP response (decrease) post-baseline compared with patients treated with placebo (ramucirumab: 35.4% vs. placebo: 9.3%; *p* < 0.0001) and were less likely to experience AFP progression (increase) at any time post-baseline compared with those treated with placebo (ramucirumab: 62.0% vs. placebo: 76.1%; *p* = 0.0005). The median time to AFP response among AFP responders in the ramucirumab arm was 1.1 months.

**Fig. 4:**
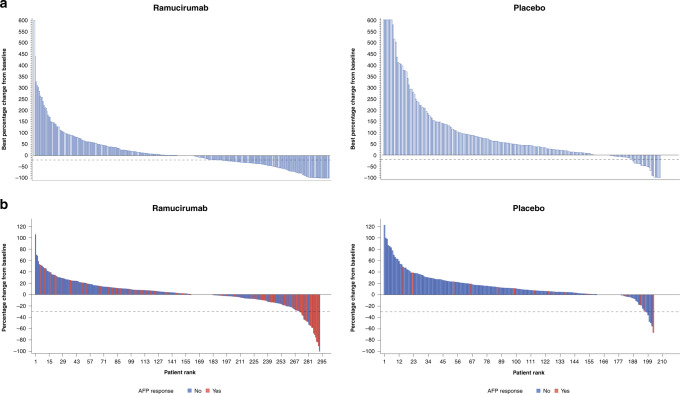
Best percentage change in AFP and radiographic tumour response by treatment arm. Waterfall plots of response for patients by treatment arm in the pooled population of patients with AFP ≥ 400 ng/ml in the REACH-2 and REACH studies: **a** the best percentage change in AFP from baseline measurements by treatment arm and b best percentage change in radiographic tumour response and relationship with AFP response. AFP alpha-fetoprotein.

Waterfall plots of the best percentage change in AFP from baseline for patients treated with ramucirumab or placebo also support the results of the analyses on AFP response and progression (Fig. 4a). The proportion of patients who experienced an increase in AFP was lower in the ramucirumab arm; the magnitude of the increase also appeared smaller when compared with the placebo arm.

### Radiographic response or progression

In the pooled population of patients with AFP ≥ 400 ng/ml in the REACH-2 and REACH studies, the median time to radiographic progression was 2.8 months in the ramucirumab arm and 1.5 months in the placebo arm (HR 0.549; *p* < 0.0001) (Fig. 3b).

Waterfall plots of radiographic tumour response by treatment arm and the relationship with AFP response are shown in Fig. 4b. A higher proportion of patients experienced a radiographic response in the ramucirumab arm compared with the placebo arm. Most (14/17) patients in the ramucirumab group and only one patient (1/2) in the placebo group with a radiographic response also experienced an AFP response.

For the first 6 weeks, AFP progression was recorded in 64% of patients with radiological progression, compared with 26% of patients without radiological progression (Supplementary Table 4). For weeks 6–12, 40% of patients with radiological progression and 27% of patients without radiological progression had AFP progression. A high association between AFP progression and radiographic progression occurring within each tumour assessment period was observed (OR 5.1; 95% CI, 3.2–8.1; *p* < 0.0001 for up to week 6; OR 1.8; 95% CI, 1.2–2.8, *p* = 0.0065 for weeks 6–12). As the median time to radiographic progression was 2.8 months in the ramucirumab arm and 1.5 months in the placebo arm, and AFP was assessed every 6 weeks, no association assessment was conducted for weeks 12–18.

**Fig. 5:**
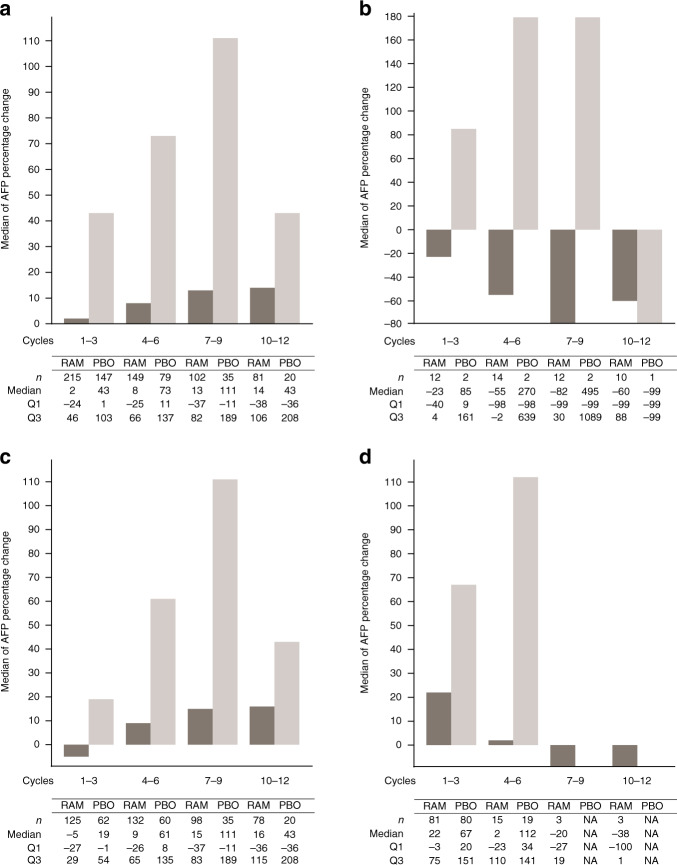
AFP percentage change from baseline by cycle. Medians of AFP percentage changes from baseline were plotted every three cycles for the pooled population of patients with AFP ≥ 400 ng/ml in the REACH-2 and REACH studies by treatment arm: **a** for all patients, **b** for patients with the best overall response of CR/PR, **c** for patients with the best overall response of CR/PR/SD and d) for patients with the best overall response of PD. AFP alpha-fetoprotein, CR complete response, ITT intent to treat, PBO placebo, PD progressive disease, PR partial response, RAM ramucirumab, SD stable disease.

In addition to assessment in all patients (Fig. 5a), the median percentage change in AFP was further evaluated in subgroups of patients defined by their best overall radiographic response (objective response [CR/PR], disease control [CR/PR/SD] and PD). For patients with a best overall radiographic response of CR/PR, the observed median percentage change from baseline in AFP decreased in the ramucirumab arm for all cycles and increased in the placebo arm (apart from one patient who received placebo for 10–12 cycles) (Fig. 5b).

For patients with a best overall response of disease control (CR/PR/SD), the median percentage AFP increase from baseline for patients in the ramucirumab arm was lower than that observed in the placebo arm at each cycle (Fig. 5c). In patients experiencing a best response of PD defined by radiographic progression, AFP increase from baseline for patients in the ramucirumab arm was lower than that observed in the placebo arm in cycles 3 and 6 (Fig. 5d). In cycles 9 and 12, AFP decreased in the ramucirumab arm, but there were no data available for placebo as most patients with a best response of progression had already discontinued treatment.

Analyses of per-patient AFP kinetics during treatment showed that AFP levels increased in both treatment arms at PD relative to AFP nadir, with a lower median percentage change in the ramucirumab arm (42.6%; interquartile range [IQR] −4.4, 171.8) compared with placebo (113.8%; IQR 27.4, 205.6) (Supplementary Fig. S4). In ramucirumab-treated patients, AFP levels were similar to baseline at AFP nadir and increased relative to nadir at the time of disease progression (Supplementary Figure S4a). Among those in the ramucirumab arm with a best radiographic response of CR/PR, a decrease was observed in AFP levels relative to baseline at the time of AFP nadir. Those with SD or PD had AFP levels similar to baseline at AFP nadir. Median AFP levels increased at the time of disease progression relative to AFP nadir, irrespective of best radiographic response achieved. In the placebo treatment arm, no reduction in AFP was observed, and AFP level increased relative to AFP nadir at the time of disease progression, irrespective of best overall response category (Supplementary Fig. S4b).

A higher proportion of patients with an AFP response received systemic anticancer therapy during follow-up compared with those without an AFP response (Supplementary Table 5). In patients treated with ramucirumab, the rate of grade 3–5 treatment-emergent adverse events was higher in those with than in those without an AFP response (Supplementary Table 6).

## Discussion

Serum AFP is widely used in clinical practice for diagnosis, pretreatment prognosis, predicting survival after transarterial chemoembolisation and tumour response to therapy, as it is considered to continuously reflect HCC tumour activity and viable burden.^24,26–33^ Our analysis of subjects from the two Phase 3 REACH and REACH-2 trials demonstrated that baseline AFP is an important prognostic factor and a predictive biomarker for ramucirumab OS benefit, and confirms that AFP ≥ 400 ng/ml is an appropriate selection criterion for ramucirumab in patients with advanced previously treated HCC.

Mechanisms of AFP overexpression and the biological characteristics of patients with tumours with high levels of AFP are not fully understood. A recent study that analysed the molecular profiles of 520 HCC patients from two independent cohorts confirmed the aberrant tumour overexpression of AFP in patients with serum concentrations >400 ng/ml, and proposed that the inverse correlation observed between AFP promoter methylation and AFP expression may play a key role in such overexpression.^34^ AFP-high tumours were characterised by poor differentiation, enrichment of progenitor features and enhanced proliferation, characteristics consistent with the prognostic capacity of AFP and the increased proportion of tumours that have serum AFP concentrations >400 ng/ml observed with disease progression. In particular, it was suggested that the significant activation of VEGF signalling displayed by AFP-high tumours could provide the rationale for the efficacy of ramucirumab in this subpopulation of HCC patients.

Additional translational work has indicated that tumours expressing AFP could represent a unique HCC subclass (S2) associated with poor prognosis, more stem cell-like features (such as EpCAM expression), increased VEGF pathway activity and increased activity of VEGFR-2-targeted antibodies in preclinical models.^20,35,36^ Ramucirumab specifically binds to VEGFR-2 and inhibits ligand-stimulated activation of its downstream intracellular signalling components, including extracellular signal-regulated kinase 1 and 2, neutralising ligand-induced proliferation and migration of human endothelial cells. Therefore, the increase in VEGF/VEGFR-2 activity in the S2 subtype of HCC, associated with elevated AFP, may be more responsive to agents that inhibit the VEGF pathway, including ramucirumab. A prospective study to validate this link is needed.

In REACH, a consistent ramucirumab treatment benefit compared with placebo was demonstrated for advanced HCC patients with AFP ≥ 400 ng/ml, whereas no meaningful OS treatment effect was observed in patients with AFP < 400 ng/ml. On the basis of these results, single-agent ramucirumab was evaluated in the second-line treatment of HCC patients with an elevated AFP level (≥400 ng/ml) in REACH-2 and was shown to significantly improve OS and PFS. This was the first positive Phase 3 study validating this approach of selecting a biomarker-enriched patient population with advanced HCC.

Additional analyses presented herein are supportive that ≥400 ng/ml for baseline AFP is an appropriate selection criterion for ramucirumab in patients with previously treated HCC. In the STEPP analysis for patients in REACH, the OS HR fluctuated > and <1.0 for patients with median baseline AFP < 400 ng/ml, but was consistently <1.0 for patients with AFP ≥ 400 ng/ml, suggesting a ramucirumab treatment benefit compared with placebo for AFP level ≥400 ng/ml. In REACH-2 (which included only patients with AFP ≥ 400 ng/ml), the STEPP analysis showed a consistent OS benefit in all AFP subgroups, with point estimates of the HRs consistently <1.0. In addition, the OS HRs favoured treatment benefit with ramucirumab for all baseline AFP quartile subgroups in the pooled population of patients with AFP ≥ 400 ng/ml in the REACH-2 and REACH studies. For interpretation, it is important to note that REACH and REACH-2 were not designed to show a statistically significant treatment difference in patient subgroups. Nevertheless, taken together, the STEPP analyses of REACH and REACH-2 and the associated analyses of AFP by quartile demonstrate a consistent OS benefit with ramucirumab in all patients with AFP ≥ 400 ng/ml.

A decline in serum AFP levels during treatment has been associated with tumour response in HCC patients who received various other systemic therapies. In a secondary analysis of the CELESTIAL trial, the tyrosine kinase inhibitor, cabozantinib, was associated with improved TTP, greater rates of target lesion regression and AFP response (defined as a decrease of ≥20% in AFP level from baseline at week 8) compared with placebo in 470 patients with previously treated advanced HCC.^37^ Similarly, in an exploratory analysis of the RESORCE study, an AFP response (defined as a decrease of ≥20% in AFP level from baseline at the start of cycle 3) with the multikinase inhibitor, regorafenib, was associated with improved OS in 232 patients with HCC.^38^ Reductions in AFP levels have also been shown to be associated with treatment outcomes of immune checkpoint inhibitors in patients with advanced HCC. Among 43 patients who received immune checkpoint inhibitor therapy in clinical trials for advanced HCC at a medical referral centre in Taiwan, early AFP response (defined as >20% decline in serum AFP levels within the first 4 weeks of treatment initiation) was associated with higher treatment efficacy.^39^ AFP responders exhibited significantly longer OS (median, 28.0 vs. 11.2 months) and PFS (median, 15.2 vs. 2.7 months) compared with AFP non-responders. These differences in survival outcomes remained after adjusting for multiple variables, including various treatments.

Consistent with prior studies, we observed that on-treatment changes in AFP levels in the pooled population of patients with AFP ≥ 400 ng/ml in REACH and REACH-2 were associated with radiographic TTP and OS.^25^ Ramucirumab prolonged both time to AFP progression and radiographic TTP and appeared to slow the rate of AFP increase during treatment compared with placebo. More patients receiving ramucirumab in the pooled REACH/REACH-2 population with AFP ≥ 400 ng/ml experienced both an AFP and a radiographic response compared with placebo, and more patients had stable AFP or radiographic SD with ramucirumab than with placebo. Even in patients who only experienced AFP or radiographic progression, the amplitude of the observed AFP or tumour increase was generally lower. Additional analyses of per-patient changes in local AFP levels from baseline to TTP also support an association between AFP and radiographic disease status. Taken together, our data suggest an association between a decrease in AFP with tumour response and an increase in AFP level with disease progression. Radiographic response and any subsequent impact on AFP level can be attributed to the treatment effect of ramucirumab on HCC. The observation that median percentage change in AFP levels in the ramucirumab arm are slightly lower than those in the placebo arm at TTP is likely related to a ramucirumab treatment effect, as opposed to an interaction between ramucirumab and AFP assessment.

Certain limitations must be considered in exploratory analyses such as these. These results were demonstrated in a subpopulation well defined by the inclusion and exclusion criteria of the REACH and REACH-2 studies, and so are limited to Child-Pugh A patients who had received prior sorafenib. Furthermore, in the quartile analyses, patient numbers and events in individual quartiles are relatively small. Prospective studies are required to confirm the utility of assessing changes in AFP over time, and to select the optimal AFP decrease cut-off, to predict OS in patients with HCC.

## Conclusions

Evaluation and validation of predictive biomarkers for selected patient subgroups in early clinical trial settings could avoid trial failure in later stages of clinical development. Our findings support the prognostic impact of baseline AFP as an important factor to consider in trial design. The current analyses also suggest that decreases in AFP may be predictive of ramucirumab OS benefit and confirm AFP ≥ 400 ng/ml as an appropriate selection criterion for ramucirumab OS benefit.

## Supplementary information

Supplementary materials

## Data Availability

Eli Lilly and Company provides access to all individual participant data collected during the trial, after anonymisation, with the exception of pharmacokinetic or genetic data. Data are available to request 6 months after the indication studied has been approved in the United States and EU and after primary publication acceptance, whichever is later. No expiration date of data requests is currently set once data are made available. Access is provided after a proposal has been approved by an independent review committee identified for this purpose and after receipt of a signed data-sharing agreement. Data and documents, including the study protocol, statistical analysis plan, clinical study report, blank or annotated case report forms, will be provided in a secure data-sharing environment. For details on submitting a request, see the instructions provided at www.vivli.org.
