# Supplementary material for: Serum alpha-fetoprotein and clinical outcomes in patients with advanced hepatocellular carcinoma treated with ramucirumab
Source: Br J Cancer. 2021 Feb 3;124(8):1388–97. doi: 10.1038/s41416-021-01260-w (PMC8039038; doi:10.1038/s41416-021-01260-w)
Supplement: Supplementary file 1 — Supplementary materials [file 41416_2021_1260_MOESM1_ESM.docx]

**Prognostic and predictive value of baseline** **alpha-fetoprotein in patients with advanced hepatocellular carcinoma treated with ramucirumab**

Andrew X. Zhu, Richard S. Finn, Yoon-Koo Kang, Chia-Jui Yen, Peter R. Galle, Josep M. Llovet, Eric Assenat, Giovanni Brandi, Kenta Motomura, Izumi Ohno, Bruno Daniele, Arndt Vogel, Tatsuya Yamashita, Chih-Hung Hsu, Guido Gerken, John Bilbruck, Yanzhi Hsu, Kun Liang, Ryan C. Widau, Chunxiao Wang, Paolo Abada and Masatoshi Kudo

**SUPPLEMENTARY INFORMATION**

**Supplementary Fig. S1 Modelled relationship between a specific baseline AFP value and the expected OS HR based on an unstratified Cox model – REACH.**[**^17^**](#_ENREF_17) *AFP* alpha-fetoprotein; *HR* hazard ratio


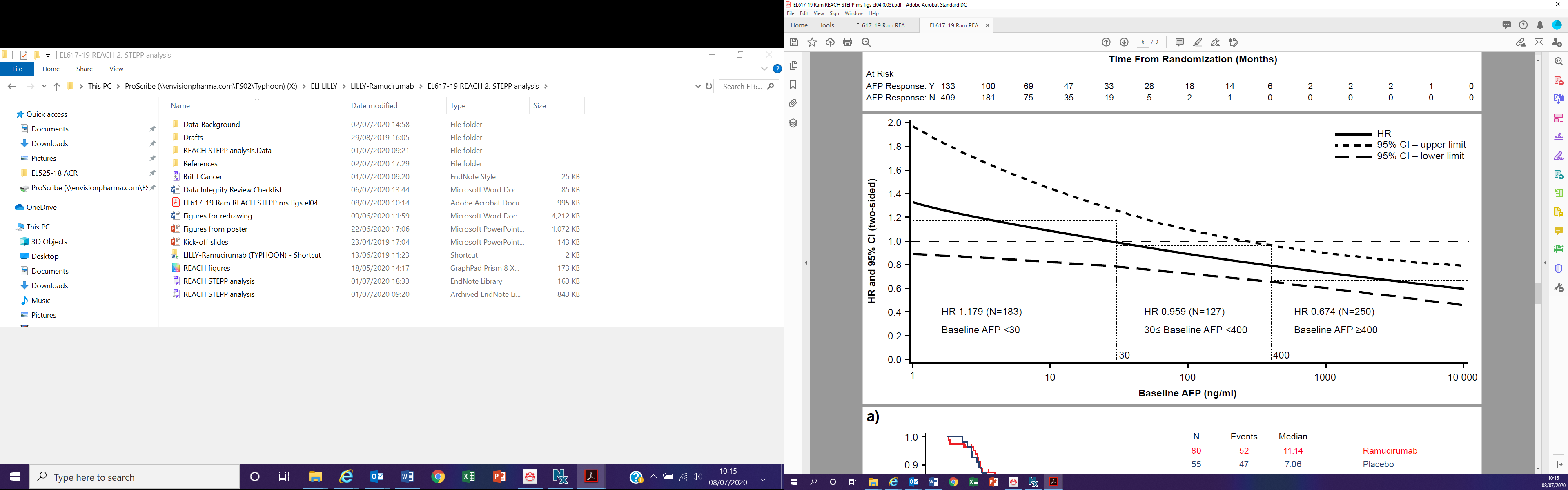


**Supplementary Fig. S2** **Kaplan-Meier graph of OS in the pooled population of patients with AFP ≥400 ng/ml in the REACH-2 and REACH studies for a) baseline AFP Quartile 1 (<1236.9 ng/ml), b) baseline AFP Quartile 2 (1236.9 ng/ml to <4081.5 ng/ml), c) baseline AFP Quartile 3 (4081.5 ng/ml to <22,000 ng/ml) and d) baseline AFP Quartile 4. (≥22,000 ng/ml)** *HR* hazard ratio; *OS* overall survival.


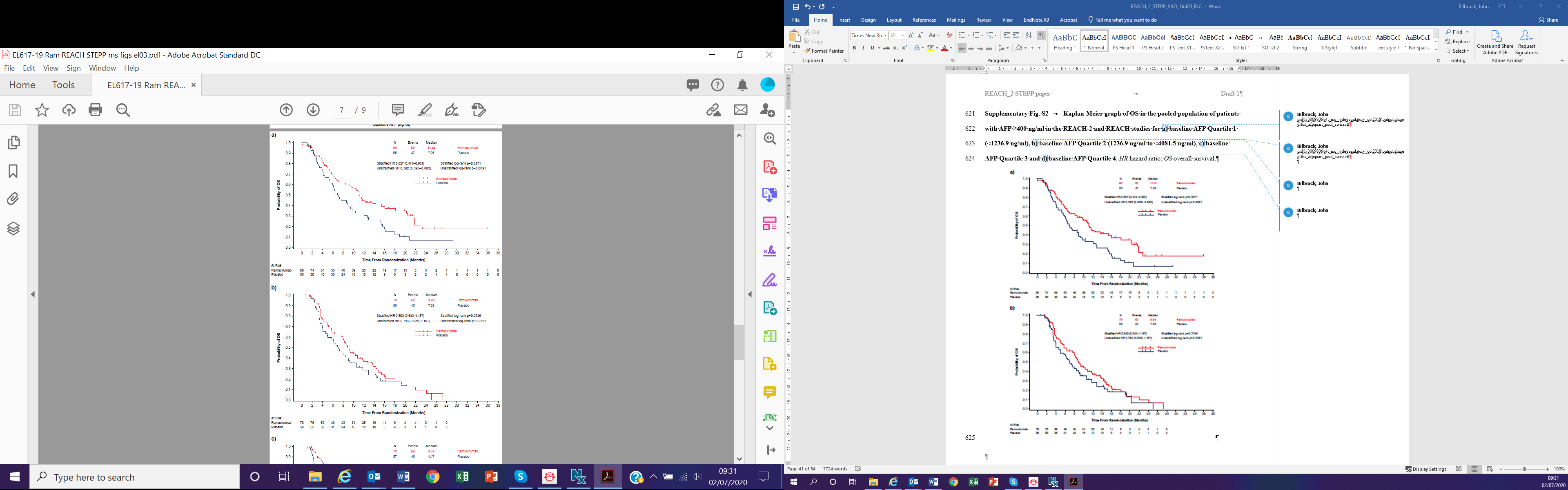


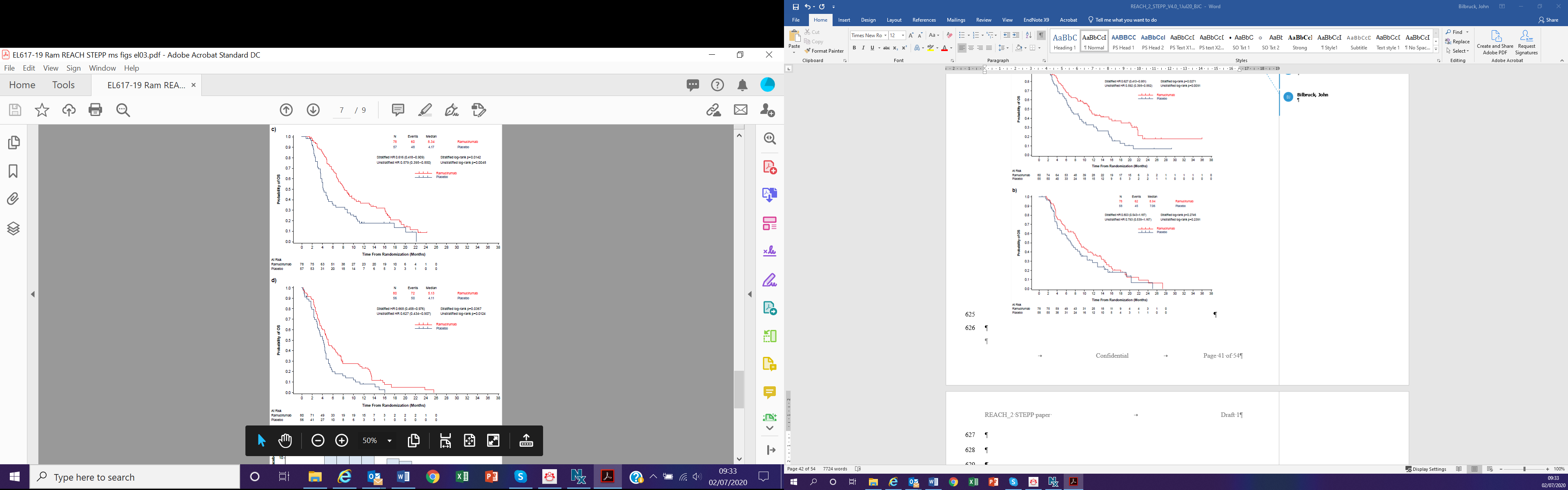


**Supplementary Fig. S3 Baseline AFP distribution in patients in the pooled population of patients with AFP ≥400 ng/ml in the REACH-2 and REACH studies.** *AFP* alpha-fetoprotein.


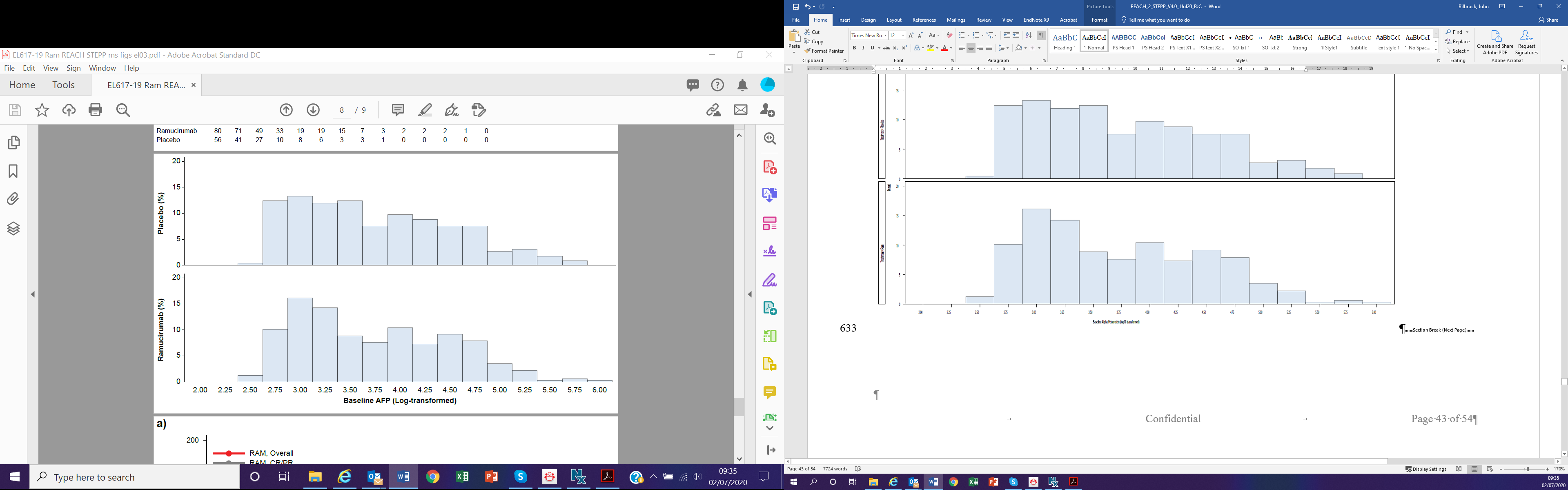


**Supplementary Fig. S4** **AFP percentage change from baseline to nadir and from nadir to PD by best radiographic response in the pooled population of patients with AFP ≥400 ng/ml in the REACH-2 and REACH studies: a) ramucirumab treatment arm and b) placebo treatment arm.** *AFP* alpha-fetoprotein; *CR* complete response; *PD* progressive disease; *PR* partial response; *RAM* ramucirumab; *SD* stable disease.


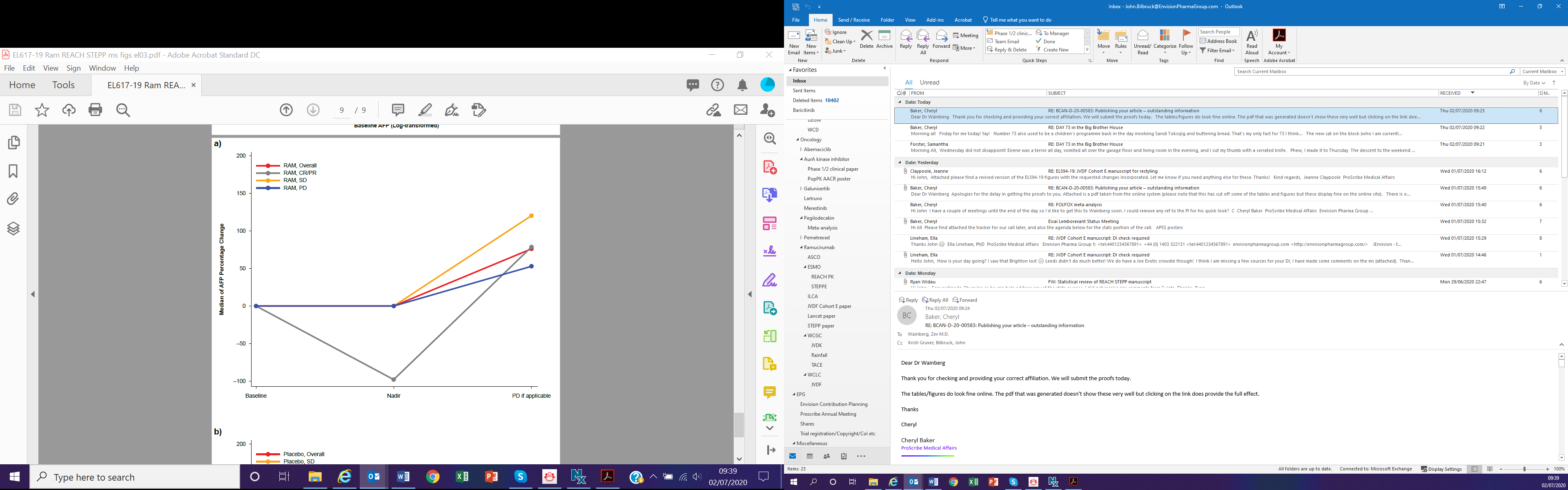


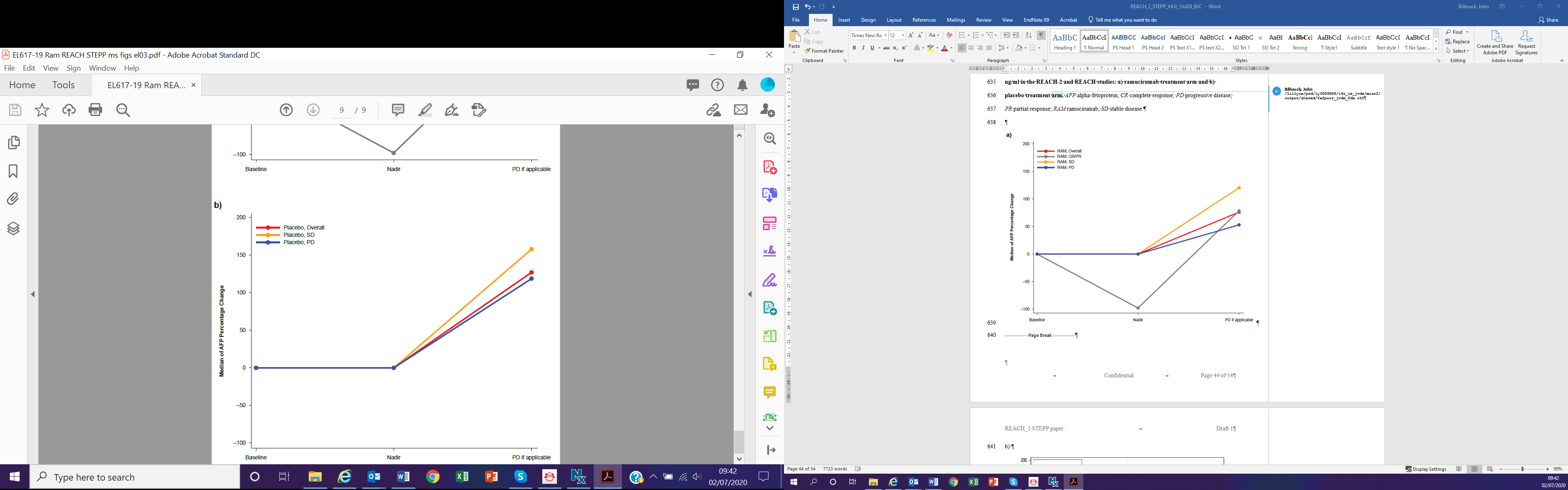


**Supplementary Table 1.** OS by baseline AFP quartiles for pooled population of patients with AFP ≥400 ng/ml in the REACH-2 and REACH studies.

| Quartile (Q) | AFP concentration (ng/ml) | Number of patients/events | | Median OS (months) | | Hazard ratio (95% CI) |
| --- | --- | --- | --- | --- | --- | --- |
|  |  | Ramucirumab | Placebo | Ramucirumab | Placebo |  |
| Overall  Q1  Q2  Q3  Q4 | –  <1236.9  1236.9–<4081.5  4081.5–<22 000  ≥22 000 | 316/246  80/52  78/62  78/60  80/72 | 226/190  55/47  58/45  57/48  56/50 | 8.1  11.1  8.9  8.3  5.1 | 5.0  7.1  7.1  4.2  4.1 | 0.694 (0.571–0.842)  0.627 (0.413–0.951)  0.803 (0.543–1.187)  0.616 (0.418–0.909)  0.668 (0.458–0.976) |

*AFP* alpha-fetoprotein; *OS* overall survival.

**Supplementary Table 2.** Baseline and disease characteristics by AFP response in pooled population of patients with AFP ≥400 ng/ml in the REACH-2 and REACH studies**.**

|  | Ramucirumab | | Placebo | |
| --- | --- | --- | --- | --- |
|  | AFP response  (N=112) | No AFP response  (N=204) | AFP response  (N=21) | No AFP response  (N=205) |
| Age, years, mean (standard deviation)  Male, n (%)  Race, n (%)  White  Asian  Other/Missing  ECOG PS, n (%)  0  1  Median duration of disease (range), months  Child-Pugh score, n (%)  A-5  A-6  B-7  B-8  Barcelona Clinic Liver Cancer, n (%)  Stage B  Stage C  Aetiology of liver disease, n (%)  Hepatitis B virus  Hepatitis C virus  Significant alcohol abuse  Steatohepatitis  Haemochromatosis  Other  Macrovascular invasion present, n (%)  Yes  No  Extrahepatic spread present, n (%)  Yes  No  Median duration of prior sorafenib (range), months  Time from last sorafenib treatment, ≥1 month, n (%)  Median AFP (IQR), ng/ml | 64.9 (10.2)  93 (83.0)  47 (42.0)  45 (40.2)  20 (17.9)  57 (50.9)  55 (49.1)  20.2 (2.9–259.7)  63 (56.3)  49 (43.8)  0  0  19 (17.0)  93 (83.0)  32 (28.6)  34 (30.4)  26 (23.2)  9 (8.0)  2 (1.8)  17 (15.2)  42 (37.5)  70 (62.5)  76 (67.9)  36 (32.1)  3.8 (0.4–44.4)  56 (50.0)  3591 (1170–20 205) | 62.1 (11.3)  153 (75.0)  63 (30.9)  123 (60.3)  18 (8.8)  116 (56.9)  88 (43.1)  18.0 (0.2–180.2)  127 (62.3)  73 (35.8)  1 (0.5)  3 (1.5)  26 (12.7)  178 (87.3)  92 (45.1)  49 (24.0)  45 (22.1)  17 (8.3)  0  26 (12.7)  71 (34.8)  133 (65.2)  150 (73.5)  54 (26.5)  3.5 (0.5–56.3)  90 (44.1)  4473 (1313–23 217) | 62.8 (12.6)  17 (81.0)  12 (57.1)  8 (38.1)  1 (4.8)  10 (47.6)  11 (52.4)  18.0 (4.4–160.1)  11 (52.4)  10 (47.6)  0  0  2 (9.5)  19 (90.5)  6 (28.6)  9 (42.9)  5 (23.8)  2 (9.5)  0  2 (9.5)  7 (33.3)  14 (66.7)  15 (71.4)  6 (28.6)  4.3 (0.5–36.0)  10 (47.6)  1853 (795–8564) | 60.8 (11.8)  172 (83.9)  68 (33.2)  115 (56.1)  22 (10.7)  108 (52.7)  97 (47.3)  14.3 (0.1–160.1)  124 (60.5)  79 (38.5)  2 (1.0)  0  27 (13.2)  178 (86.8)  96 (46.8)  47 (22.9)  38 (18.5)  9 (4.4)  1 (0.5)  28 (13.7)  70 (34.1)  135 (65.9)  156 (76.1)  49 (23.9)  3.9 (0.5–36.5)  84 (41.0)  4430 (1393–23 529) |

*AFP* alpha-fetoprotein; *ECOG PS* Eastern Cooperative Oncology Group performance status; *IQR* interquartile range.

**Supplementary Table 3.** Treatment duration in pooled population of patients with AFP ≥400 ng/ml in the REACH-2 and REACH studies.

|  | Ramucirumab | | Placebo | |
| --- | --- | --- | --- | --- |
|  | AFP response  (N=112) | No AFP response  (N=204) | AFP response  (N=21) | No AFP response  (N=202) |
| Median duration of therapy (IQR), weeks  Median number of cycles (IQR)  Median relative dose intensity (IQR), % | 19.4 (8.0–41.9)  9 (4–19.5)  97.4 (91.7–100) | 8.0 (6.0–14.0)  4 (3–7)  98.7 (95.4–100.3) | 14.6 (8.0–33.7)  7 (4–17)  97.7 (95.2–100.3) | 6.1 (5.9–12.0)  3 (3–6)  99.8 (97.9–101.0) |

*AFP* alpha-fetoprotein; *IQR* interquartile range.

**Supplementary Table 4.** Radiographic progression and AFP progression by tumour measurement period in pooled population of patients with AFP ≥400 ng/ml in the REACH-2 and REACH studies**.**

|  |  | Patients with radiographic progression | Patients without radiographic progression | p-value^a^ | Odds ratio^b^  (95% CI) |
| --- | --- | --- | --- | --- | --- |
| Up to 6 weeks | N | 97 | 445 | <0.0001 | 5.08 (3.19−8.10) |
| AFP progression | n (%) | 62 (63.9) | 115 (25.8) |  |  |
| No AFP progression | n (%) | 35 (36.1) | 330 (74.2) |  |  |
| 6–12 weeks | N | 171 | 219 | 0.0065 | 1.83 (1.20−2.81) |
| AFP progression | n (%) | 69 (40.4) | 59 (26.9) |  |  |
| No AFP progression | n (%) | 102 (59.6) | 160 (73.1) |  |  |

*AFP* alpha-fetoprotein.
^a^p-value from Fisher’s exact test comparing AFP categories (AFP event, no event) between two TTP groups within each time period.

^b^Odds ratio for AFP event vs. no AFP event comparing between the two TTP groups within each time period.

**Supplementary Table 5.** Summary of post-discontinuation therapy by AFP response in pooled population of patients with AFP ≥400 ng/ml in the REACH-2 and REACH studies.

| Post-discontinuation therapy, n (%) | Ramucirumab | | Placebo | |
| --- | --- | --- | --- | --- |
|  | AFP response  (N=112) | No AFP response  (N=204) | AFP response  (N=21) | No AFP response  (N=205) |
| Systemic therapy overall  Chemotherapy  Immunotherapy/Immunomodulatory  Targeted antibody/small molecule  Investigational drug/Randomised trial | 39 (34.8)  15 (13.4)  10 (8.9)  15 (13.4)  4 (3.6) | 57 (27.9)  34 (16.7)  3 (1.5)  23 (11.3)  4 (2.0) | 6 (28.6)  4 (19.0)  1 (4.8)  0  0 | 53 (25.9)  32 (15.6)  6 (2.9)  14 (6.8)  2 (1.0) |

*AFP* alpha-fetoprotein.

**Supplementary Table 6.** Summary of grade 3/4/5 treatment-emergent adverse events by AFP response in pooled population of patients with AFP ≥400 ng/ml in the REACH-2 and REACH studies.

| TEAE, n (%) | Ramucirumab | | Placebo | |
| --- | --- | --- | --- | --- |
|  | AFP response  (N=112) | No AFP response  (N=204) | AFP response  (N=21) | No AFP response  (N=202) |
| Any grade 3/4/5  Aspartate aminotransferase increased  Hypertension  Blood bilirubin increased  Anaemia  Tumour pain  Alanine aminotransferase increased  Malignant neoplasm progression  Hyperbilirubinaemia  Hyponatraemia  Ascites | 74 (66.1)  5 (4.5)  21 (18.8)  4 (3.6)  6 (5.4)  0  1 (0.9)  3 (2.7)  1 (0.9)  8 (7.1)  6 (5.4) | 107 (52.5)  10 (4.9)  17 (8.3)  6 (2.9)  6 (2.9)  1 (0.5)  2 (1.0)  10 (4.9)  2 (1.0)  8 (3.9)  9 (4.4) | 13 (61.9)  4 (19.0)  0  3 (14.3)  2 (9.5)  2 (9.5)  2 (9.5)  2 (9.5)  2 (9.5)  1 (4.8)  1 (4.8) | 103 (51.0)  21 (10.4)  8 (4.0)  14 (6.9)  3 (1.5)  1 (0.5)  6 (3.0)  8 (4.0)  10 (5.0)  4 (2.0)  8 (4.0) |

TEAEs that occurred at a ≥5% frequency (grade 3/4/5) in any subgroup in the overall safety population are listed.

*AFP* alpha-fetoprotein; *TEAE* treatment-emergent adverse event.
